# YB-1 regulates tumor growth by promoting MACC1/c-Met pathway in human lung adenocarcinoma

**DOI:** 10.18632/oncotarget.18262

**Published:** 2017-05-29

**Authors:** Tao Guo, Shilei Zhao, Peng Wang, Xiaoyuan Xue, Yan Zhang, Mengying Yang, Nan Li, Zhuoshi Li, Lingzhi Xu, Lei Jiang, Lei Zhao, Patrick C. Ma, Rafael Rosell, Jinxiu Li, Chundong Gu

**Affiliations:** ^1^ Department of Thoracic Surgery, The First Affiliated Hospital of Dalian Medical University, Dalian 116011, China; ^2^ Lung Cancer Diagnosis and Treatment Center of Dalian, The First Affiliated Hospital of Dalian Medical University, Dalian 116011, China; ^3^ Institute of Cancer Stem Cell, Dalian Medical University, Dalian 116044, China; ^4^ Department of Radiation Oncology, Qianfoshan Hospital Affiliated to Shandong University, Jinan 250000, China; ^5^ The Second Affiliated Hospital, Dalian Medical University, Dalian 116011, China; ^6^ The Fourth Affiliated Hospital, Anhui Medical University, Hefei 230000, China; ^7^ Aerodigestive Oncology Translational Research THOR, Department of Solid Tumor Oncology, Taussig Cancer Institute, Cleveland Clinic, Cleveland, OH 44195, USA; ^8^ Breakthrough Cancer Research Unit, Pangaea Biotech, Dexeus University Institute, Catalan Institute of Oncology, Badalona 08916, Spain Written on behalf of the AME Thoracic Surgery Collaborative Group

**Keywords:** YB-1, MACC1, c-Met, lung adenocarcinoma, prognosis

## Abstract

Aberrant overexpression of the transcription/translation factor Y-box-binding protein (YB-1) is associated with poor prognosis of lung adenocarcinoma, however the underlying mechanism by which YB-1 acts has not been fully elucidated. Here, we reported that inhibition of YB-1 diminished proliferation, migration and invasion of lung adenocarcinoma cells. Interestingly, we identified metastasis associated in colon cancer-1 (MACC1) as a target of YB-1. Depletion of YB-1 markedly decreased *MACC1* promoter activity and suppressed the MACC1/c-Met signaling pathway in lung adenocarcinoma cells. Additionally, chromatin immunoprecipitation (ChIP) assay demonstrated that YB-1 bound to the MAC*C1* promoter. Moreover, YB-1 was positively correlated with MACC1, and both proteins were over-expressed in lung adenocarcinoma tissues. The Cox-regression analysis indicated that high YB-1 expression was an independent risk factor for prognosis in enrolled patients. Furthermore, depletion of YB-1 attenuated tumorigenesis in a xenograft mouse model and reduced MACC1 expression in tumor tissues. Collectively, our data suggested that targeting YB-1 suppressed lung adenocarcinoma progression through the MACC1/c-Met pathway and that the high expression of YB-1/MACC1 is a potential prognostic marker in lung adenocarcinoma.

## INTRODUCTION

Lung cancer is the main cause of cancer death worldwide [1]. Non-small-cell lung cancer (NSCLC) accounts for approximately 85% of lung cancers, with adenocarcinoma as the most common subtype followed by squamous carcinoma [2]. Most patients with NSCLC are diagnosed at late stages, when curative treatment is not feasible due to tumor dissemination, invasion and distant metastases [3]. These facts highlight the need for better understanding of the underlying molecular pathways and mechanisms of lung cancer metastasis, in order to develop novel therapeutic approaches.

YB-1 is a multifunctional protein that regulates transcription by binding to the Y-box motif, an inverted CCAAT box, at the promoter or enhancer of target genes [4]. YB-1 plays prominent pro-oncogenic roles in tumor invasion and metastasis [5–7], drug resistance [8], cell proliferation [9] and DNA repair [10]. YB-1 promotes several genes expression, including *MET* protooncogene (also called hepatocyte growth factor receptor, HGFR) [11]. Recent studies showed that the dysregulated activation of the HGF/c-Met pathway correlates with the progression of a wide range of human cancers and is thought to contribute to EMT, tumor proliferation, invasion and metastasis [11–15]. In previous reports, we and other investigators found that high YB-1 expression in lung adenocarcinoma was correlated with poor outcomes and metastasis of lung adenocarcinoma patients [16–18]. Nevertheless, the functional pathways and molecular mechanism by which YB-1 acts in lung adenocarcinoma has not been fully elucidated.

MACC1 is a prognostic biomarker for colorectal cancer metastasis and patient survival that was recently identified in human colon cancer tissues, metastatic tissues and normal tissues [19]. Both YB-1 and MACC1 regulate the HGF/c-Met signaling pathway and induce tumor invasion and metastasis in several cancer types [19–21]. Furthermore, we previously found that both YB-1 and MACC1 were over-expressed in lung adenocarcinoma tissue, and their expression correlated with tumor metastasis in lung adenocarcinoma [16, 22, 23]. Importantly, we identified two potential binding sites of YB-1 in the *MACC1* promoter (-1860 to -1856 and -1468 to -1464). Thus, we hypothesized that YB-1 binds to the *MACC1* promoter and up-regulates MACC1 expression to promote tumor cell invasion and tumor growth.

In this study, we set out to investigate the potential role of YB-1 in the regulation of lung adenocarcinoma progression and the mechanism involved.

## RESULTS

### Inhibition of YB-1 diminishes proliferation in lung adenocarcinoma cells

**Figure 1:**
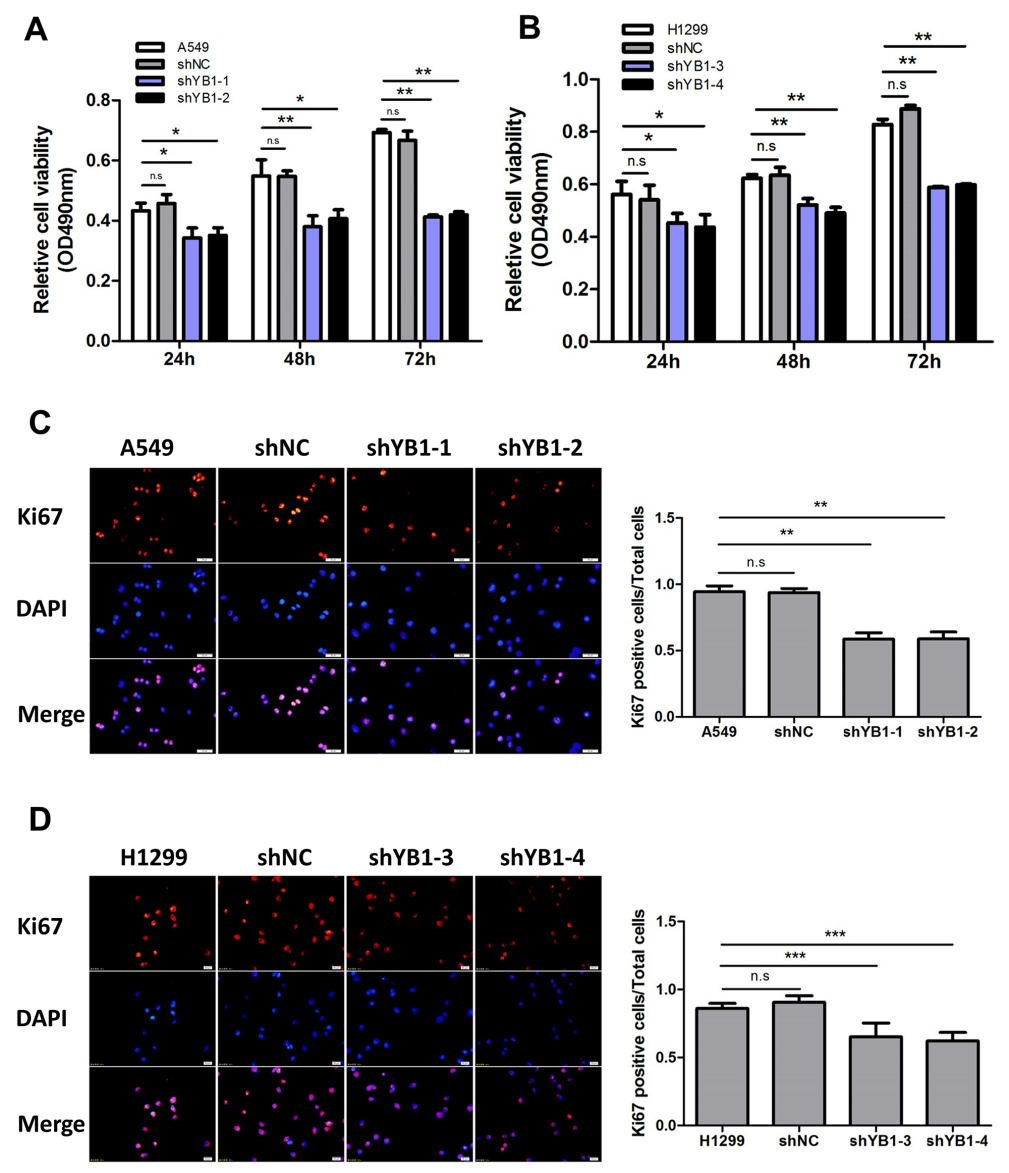
Inhibition of YB-1 diminishes proliferation of lung adenocarcinoma cells MTT assay in lung adenocarcinoma cells (**(A)**, A549 cells; **(B)**, H1299 cells). shYB1-1 and shYB1-2: YB-1-silenced A549 cells; shYB1-3 and shYB1-4: YB-1-silenced H1299 cells. **(C,D)** Immunofluorescence analysis (left panel) with corresponding summary (right panel) of Ki67 expression in lung adenocarcinoma cells (Red: Ki67; blue: DAPI). Columns were averaged from three independent experiments. * *P*<0.05, **P<0.01,*** *P*<0.005. Error bars represent mean±s.d. Scale bars, 20 μ m.

To investigate whether YB-1 expression correlates with lung adenocarcinoma progression, the stable YB-1-silenced A549 cells (shYB1-1, shYB1-2) and H1299 cells (shYB1-3, shYB1-4) were generated using two shRNA expressing plasmids (Supplementary Figure 1). The results showed that depletion of YB-1 reduced cell proliferation in both clones (Figure 1A and 1B). To determine the effects of inhibition of YB-1 on proliferation rate, we performed Ki67 immunostaining in lung adenocarcinoma cells. The proliferation rate of the shYB-1 cells was decreased compared to the control cells, as indicated by the significant decrease in the percentage of cells positive for Ki67 (Figure 1C and 1D). These data indicated that the depletion of YB-1 repressed the proliferation of lung adenocarcinoma cells.

### Inhibition of YB-1 suppresses the migration and invasion of lung adenocarcinoma cells

**Figure 2:**
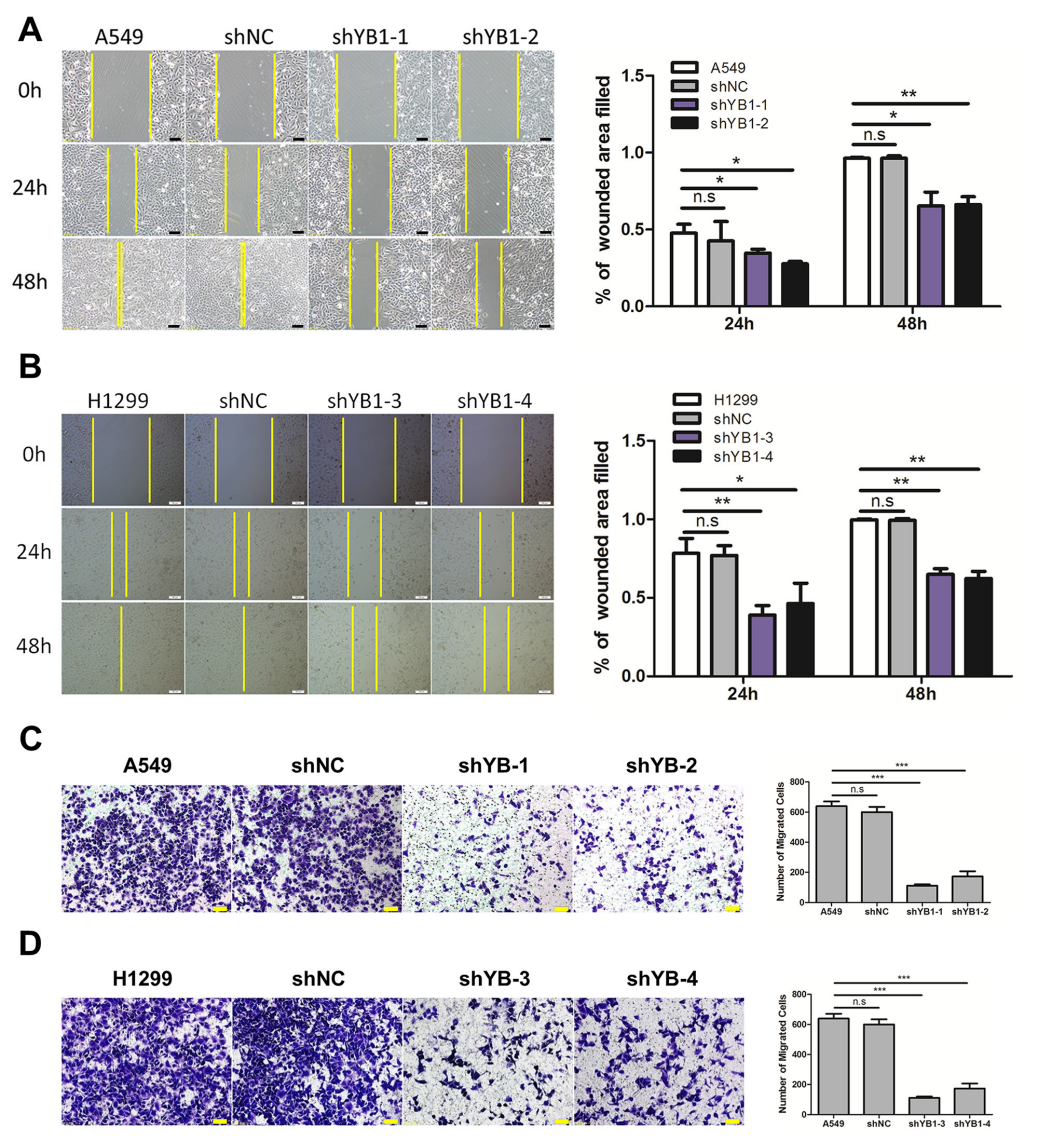
Inhibition of YB-1suppresses migration of lung adenocarcinoma cells **(A,B)** Wound healing assay of lung adenocarcinoma cells in the YB-1-silenced cells (shYB1-1 and shYB1-2: YB-1-silenced A549 cells; shYB1-3 and shYB1-4: YB-1-silenced H1299 cells) and their corresponding control cells. The distance from the wound edge was calculated with corresponding summary (right panel). **(C,D)** Transwell assay in lung adenocarcinoma cells in the YB-1-silenced cells and their corresponding control cells. Columns were averaged from three independent experiments. * *P*<0.05, **P<0.01,*** *P*<0.005, two-tailed Student's T-tests. Error bars represent mean±s.d. Scale bars, 100 μm (A,B), 50 μm (C,D).

**Figure 3:**
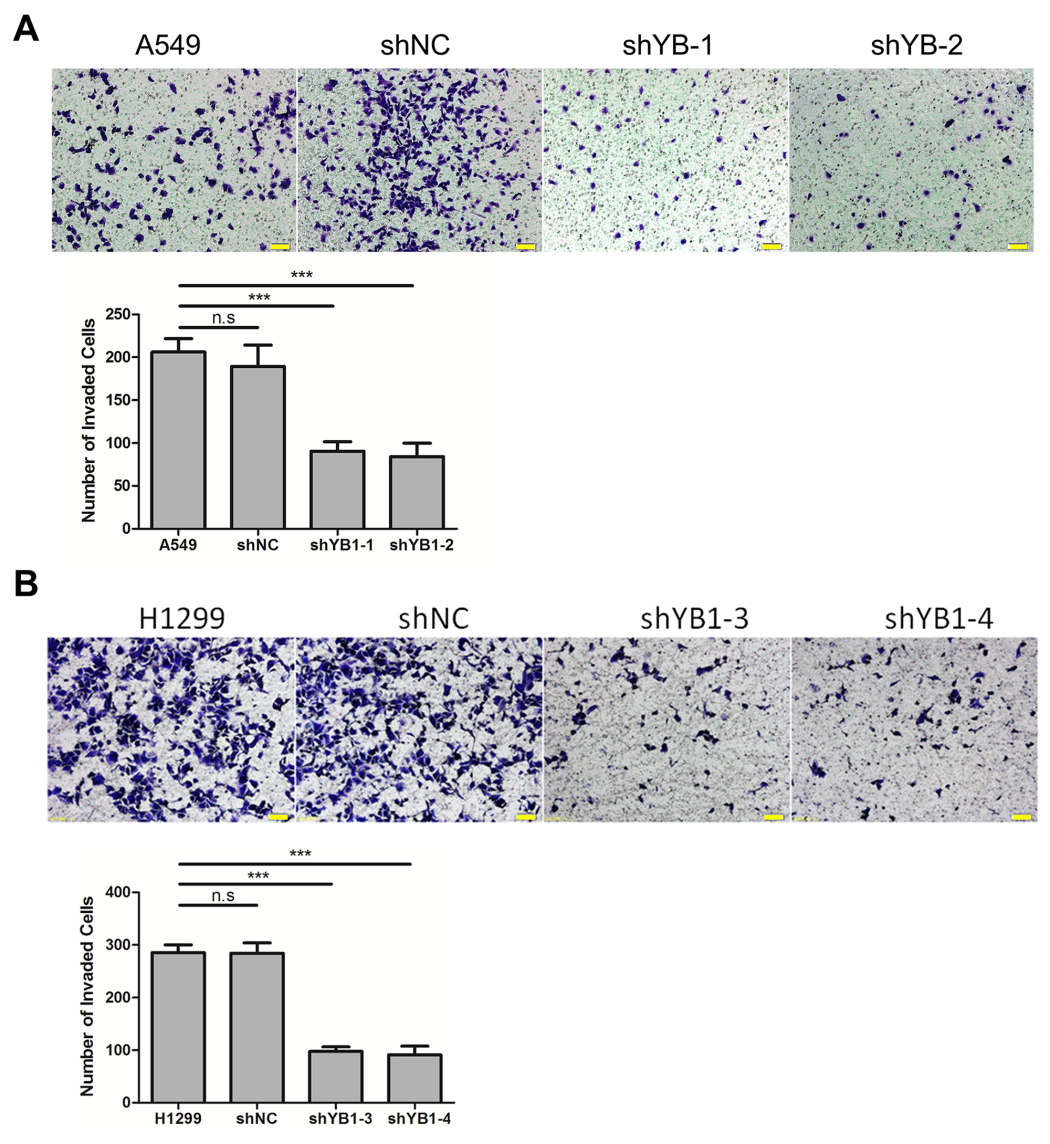
Inhibition of YB-1 suppresses invasion of lung adenocarcinoma cells The YB-1-silenced A549 cells **(A)** and H1299 cells **(B)**, and their corresponding control cells were analyzed for cell invasion across Matrigel-coated chambers (Transwell). Columns were averaged from three independent experiments. *P*<0.005. Error bars represent mean±s.d. Scale bars, 50 μm.

Next, we evaluated the effect of down-regulating YB-1 on migration and invasion in A549 and H1299 cells. Using the wound healing assay, it was observed that the YB-1-silenced cells migrated much more slowly than corresponding wild type or shNC-transfected control cells after 24 h and 48 h (Figure 2A and 2B). The results of the transwell assay showed that the migration capability of YB-1-silenced cells was reduced, compared to the corresponding control cells (Figure 2C and 2D). Similarly, depletion of YB-1 significantly diminished the invasive capability of lung adenocarcinoma cells (Figure 3A and 3B). These data showed that the inhibition of YB-1 suppressed migration and invasion in lung adenocarcinoma cells.

### YB-1 enhances *MACC1* promoter activity in lung adenocarcinoma cells

**Figure 4:**
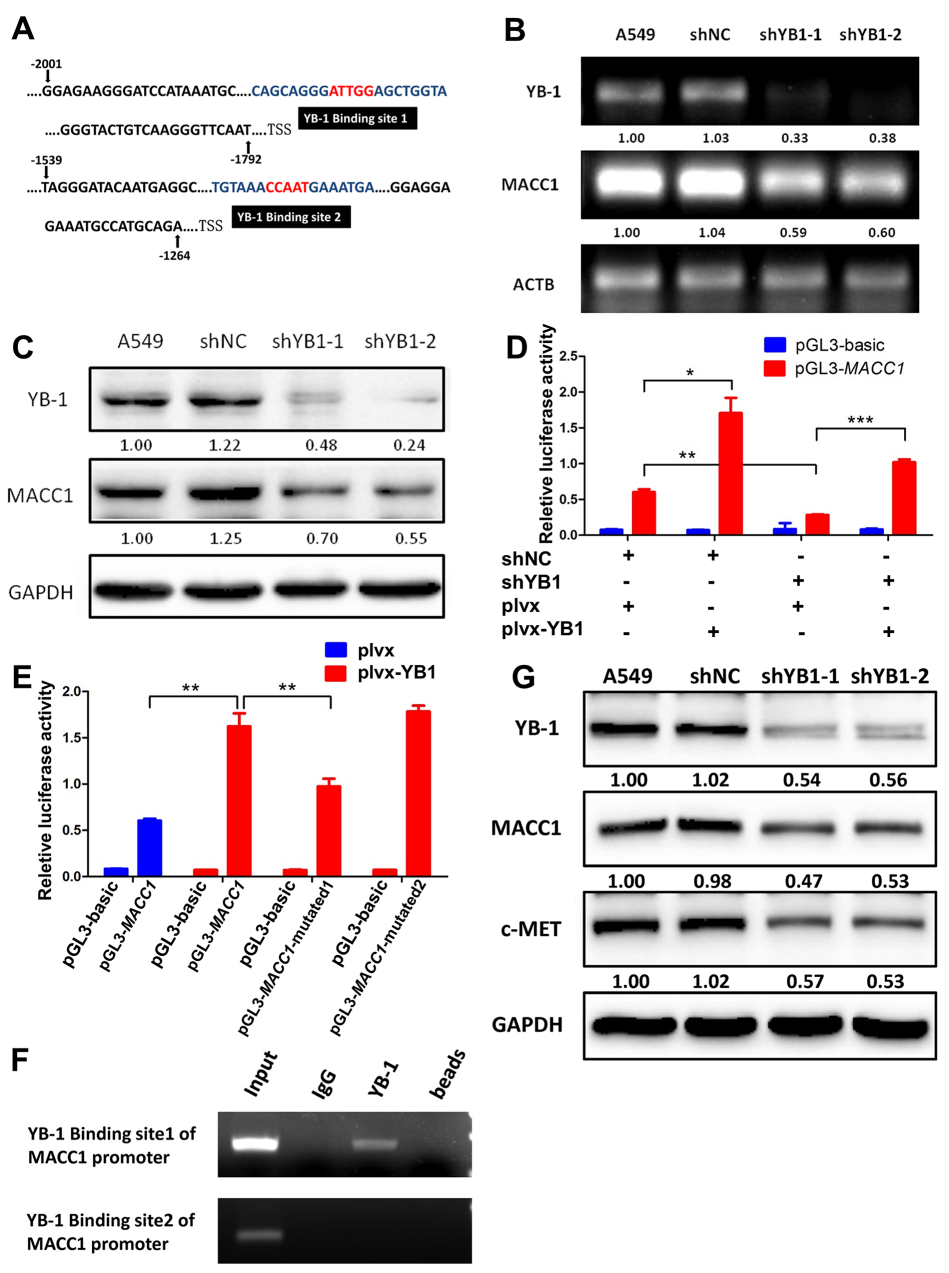
YB-1 promotes *MACC1* transcription by binding to *MACC1* promoter and activates MACC1/c-Met pathway **(A)** Analysis of the *MACC1* promoter indicated two putative YB-1 binding sites where the black boxes indicate sequences. RT-PCR analysis **(B)** and western blot analysis **(C)** determined the expression of YB-1 and MACC1 after inhibition of YB-1 in A549 cells. **(D)** *MACC1* promoter (-2020 to +262) activity was analyzed by dual-luciferase reporter assay. The YB-1-silenced A549 cells and their corresponding control cells were co-transfected with plvx control plasmid or plvx-YB1 plasmid and *MACC1* promoter (-2020 to +262) or basic reporter along with pRL-TK for 24h. **(E)** Mutated *MACC1* promoter plasmids were generated (pGL3-*MACC1*-mutated1; pGL3-*MACC1*-mutated2). YB-1 binding sites in these two plasmids were mutated at two base pairs (pGL3-*MACC1*-mutated1: -1860 to -1856; pGL3-*MACC1*-mutated2: -1468 to -1464). A549 cells were transiently co-transfected with plvx vector plasmid or plvx-YB1 plasmid and the pGL3-basic reporter, the wild type *MACC1* promoter reporter (pGL3-*MACC1*) or the mutated *MACC1* promoter plasmids (pGL3-MACC1-mutated1; pGL3-MACC1-mutated2) along with pRL-TK for 24h. Transfected cells were harvested for dual-luciferase reporter assay. **(F)** ChIP assay was performed with YB-1 antibody or non-immune IgG as negative control. Immunoprecipitated DNA was amplified by PCR using primers as indicated. **(G)** Western blot analysis of YB-1, MACC1 and c-Met expression after inhibition of YB-1 in A549 cell. * *P*<0.05, **P<0.01,*** *P*<0.005.

As both YB-1 and MACC1 promote the HGF/c-Met signaling pathway and induce tumor progression and metastasis in several cancer types [19–21], and we identified two different potential binding sites for YB-1 in the *MACC1* promoter (from -1860 to -1856; from -1468 to -1464) (Figure 4A), we postulated that YB-1 promoted cancer development through activating MACC1/ c-Met signaling pathway. Thus, we proceeded to evaluate the correlation between YB-1 and MACC1 mRNA and protein expression in lung adenocarcinoma A549 cells. Interestingly, we observed a significant decrease in *MACC1* mRNA and protein levels in YB-1-silenced cells (Figure 4B and 4C), indicating that YB-1 regulated the expression of MACC1. To verify the regulation of *MACC1* promoter by YB-1, the YB-1-silenced A549 cells and their corresponding control cells were co-transfected with plvx plasmid or plvx-YB-1 plasmid and *MACC1* promoter (-2020 to +262) reporter or basic reporter along with pRL-TK plasmid. These results showed that the activity of the *MACC1* promoter reporter was significantly elevated in plvx-YB-1 transfected A549 cells compared with plvx transfected cells. Conversely, reduced YB-1 impaired the activity of the *MACC1* promoter reporter. In addition, rescue assay showed that enforced expression of YB-1 restored the attenuated activity of the *MACC1* promoter reporter in YB-1-silenced A549 cells (Figure 4D). These results illustrated that YB-1 enhanced the transcription of *MACC1*.

Next, we generated two mutated *MACC1* promoter plasmids (pGL3-*MACC1*-mutated1; pGL3-*MACC1*-mutated2). YB-1 binding sites in these two plasmids were mutated at two base pairs (pGL3-*MACC1*-mutated1: -1860 to -1856; pGL3-*MACC1*-mutated2: -1468 to -1464). A549 cells were transiently co-transfected with plvx vector plasmid or plvx-YB-1 plasmid and the pGL3-basic reporter, the wild type *MACC1* promoter reporter (pGL3-*MACC1*) or the mutated *MACC1* promoter plasmids along with pRL-TK. As the results shown in Figure 4E, ectopic expression of YB-1 enhanced the activity of the wild type *MACC1* promoter reporter. Mutation in YB-1 binding site1 (pGL3-*MACC1*-mutated1: -1860 to -1856), but not the mutation in YB-1 binding site2 (pGL3-*MACC1*-mutated2: -1468 to -1464), reduced the elevated activity of the MACC1 promoter which was induced by ectopic expression of YB-1. These results revealed that YB-1 enhanced the transcriptional activity of *MACC1*through the YB-1 binding site 1 (-1860 to -1856) in the *MACC1* promoter.

### YB-1 binds to the *MACC1* promoter and up-regulates MACC1/c-Met pathway

To obtain direct evidence that YB-1 binds to the *MACC1* promoter, a ChIP assay was performed in A549 cells. PCR amplifications were performed using two primer sets designed to flank the potential YB-1 binding sites on the *MACC1* promoter, referred to as YB-1 binding site 1 and YB-1 binding site 2 (Figure 4A). YB-1:*MACC1* promoter binding was validated using the YB-1 binding site 1 primers, but not the YB-1 binding site 2 primers (Figure 4F). These results indicated that YB-1 directly bound to specific regions of the *MACC1* promoter (-1860 to -1856), thereby up-regulating the expression of MACC1.

The hypothesis that YB-1 activated the HGF/c-Met signaling pathway by up-regulating MACC1 also is supported by the observations that inhibition of YB-1 suppressed the expression of c-Met protein by Western blot assay (Figure 4G). Thus, these data provided evidence that YB-1 promoted MACC1 transcription by binding to the Y-box of the MACC1 promoter and thereby activating the MACC1/c-Met pathway.

### YB-1 is positively correlated with MACC1, and both proteins are over-expressed in lung adenocarcinoma cell lines and lung adenocarcinoma tissues

**Figure 5:**
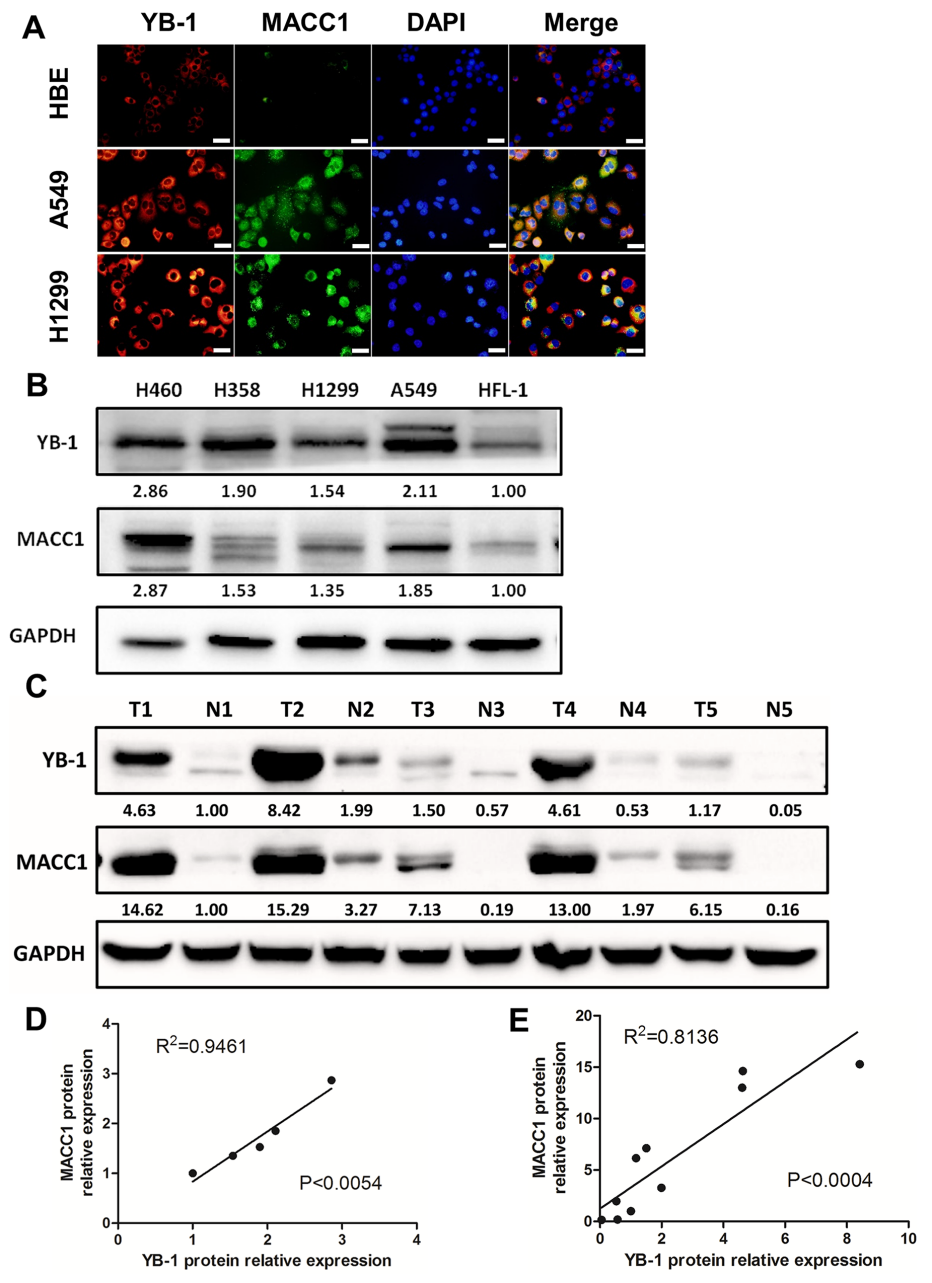
YB-1 was positively correlated with MACC1 and both proteins were over-expressed in lung adenocarcinoma cell lines **(A)** Co-localization of YB-1 and MACC1 in HBE, A549 and H1299 cells by immunofluorescence assay. Red: YB-1; Green: MACC1. Scale bars, 20 μm. Lung adenocarcinoma cell lines **(B)** and lung adenocarcinoma tissues **(C)** were lysed and subjected to Western blot to analyze YB-1 expression and MACC1 levels (T, lung adenocarcinoma tumor; N, normal lung tissues adjacent to tumor lesions). YB-1 and MACC1 protein expression levels were normalized to HFL-1 in lung adenocarcinoma cell lines. Meanwhile, those were normalized to N1 in lung adenocarcinoma tissues. Moreover, linear regression analysis was performed, **(D)** in lung adenocarcinoma ccell lines, **(E)** in lung adenocarcinoma tissues.

We explored the expression and location of YB-1 and MACC1 by immunofluorescence assay. Both YB-1 and MACC1 were elevated in human lung adenocarcinoma cell lines (A549 and H1299), compared to human bronchial epithelial cell (HBE cells), and were mainly detected in the cytoplasm in these cells (Figure 5A and Supplementary Figure 2). Subsequently, we analyzed different lung cancer cell lines and 5 lung adenocarcinoma tissue specimens by Western blot assay to verify the correlation between YB-1 and MACC1. The results showed that YB-1 and MACC1 were over-expressed in these different lung cancer cell lines, compared to human fetal lung fibroblasts (HFL-1) (Figure 5B). Linear regression analysis showed a positive correlation between YB-1 and MACC1 expression (Figure 5D, R^2^=0.9461, p<0.0054). Likewise, in the lung adenocarcinoma tissue specimens, the expression level of YB-1 and MACC1 were also much higher than in the normal lung tissues adjacent to tumor lesions (Figure 5C), and YB-1 expression was positively correlated with MACC1 expression (Figure 5E, R^2^=0.8136, p<0.0004). These data suggested that YB-1 was positively correlated with MACC1, and both proteins were over-expressed in lung adenocarcinoma cell lines and lung adenocarcinoma tissues.

### The prognostic significance of YB-1 and MACC1 for lung adenocarcinoma patients

**Figure 6:**
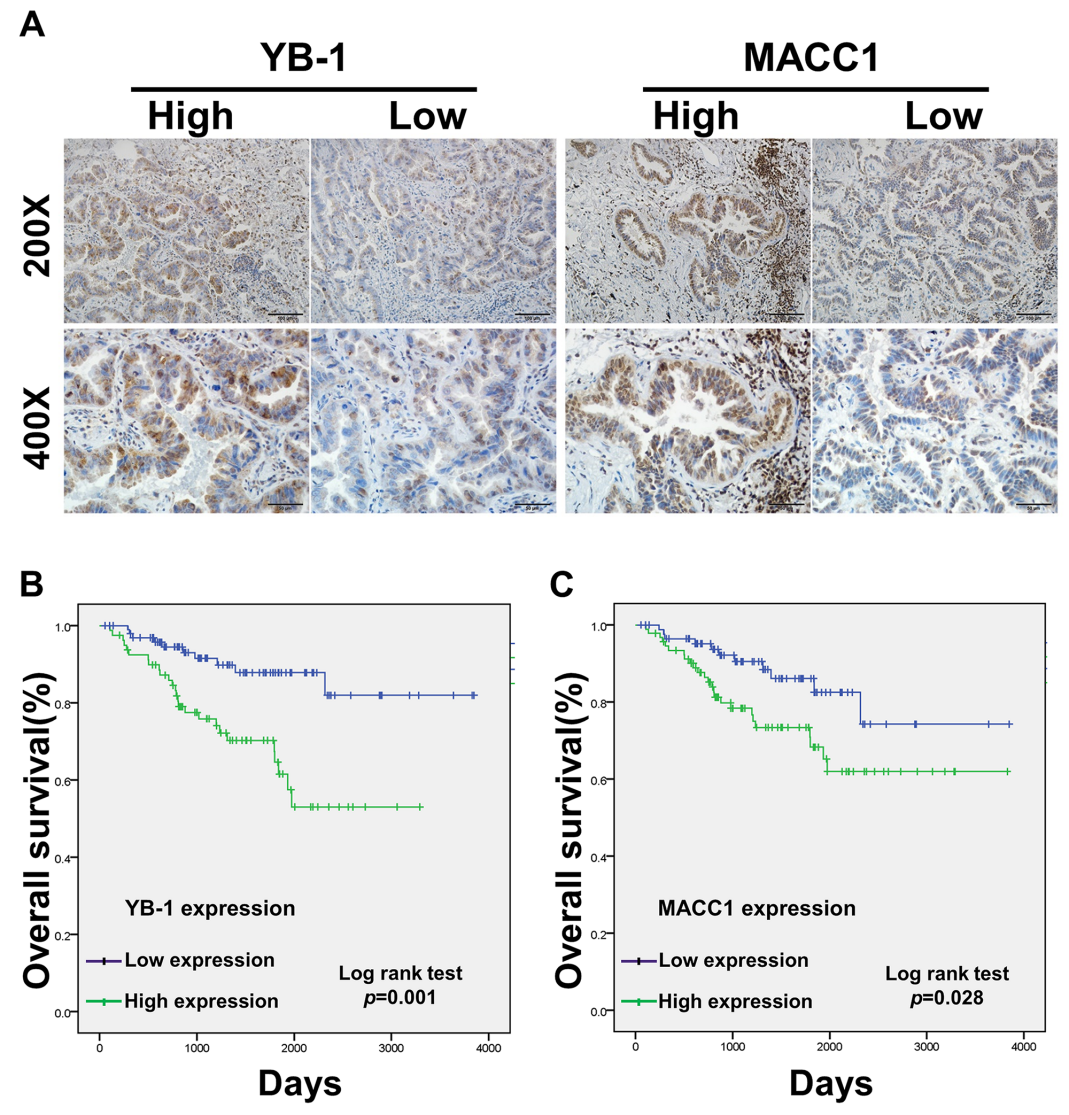
The prognostic significance of YB-1 and MACC1 for lung adenocarcinoma patients assessed via Kaplan-Meier analysis **(A)** The expression of YB-1 and MACC1 in lung adenocarcinoma specimens (n=179) was detected by IHC assay. Original magnification 200× and 400× in inset. The overall 5-year survival rates in the patients with low YB-1 expression and high YB-1 expression were 82% and 53%, respectively (p=0.004). Survival curves of low and high expression of YB-1 **(B)** and MACC1 **(C)**. For each cohort, different subgroups were plotted according to the cutoff values of YB-1 and MACC1, which were defined as the median of the cohort.

We also analyzed 179 patients with complete surgical resection of lung adenocarcinoma by immunohistochemistry (IHC) (Figure 6A). The clinical features of the patients are summarized in Table 1, the expression levels of YB-1 and MACC1 were elevated in 80 (44.7%) and 92 (51.4%) patients, respectively. The expression of YB-1 was significantly associated with TNM (TNM, tumor, node, metastases) stage (p<0.001), T state (p<0.001), lymph node metastasis (p<0.001) and differentiation (p=0.038), but not with sex and age. Additionally, MACC1 expression was related to TNM stage (p<0.001), T state (p=0.033) and lymph node metastasis (p<0.001). Furthermore, YB-1 expression was positively correlated with MACC1 expression in the 179 lung adenocarcinoma tissue specimens (Table 2 and Supplementary Figure 3).

**Table 1:** Relations between the level of YB-1 or MACC1 expression and clinicopathologic characteristics in lung adenocarcinoma

Characteristics	YB-1		P-value	MACC1		P-value
	High(%)	Low		High(%)	Low	
Over all	80(44.7)	99		92(51.4)	87	
Sex			0.761			0.489
Male	41(43.6)	53		46 (48.9)	48	
Female	39(45.9)	46		46(54.1)	39	
Age			0.791			0.831
≤69y	42(45.7)	50		48(52.2)	44	
>69y	38(43.7)	49		44(50.6)	43	
TNM stage			<0.001			<0.001
I	48(34.3)	92		60(42.9)	80	
II	13(76.5)	4		12(70.6)	5	
IIIA	16 (84.2)	3		17(89.5)	2	
IIIB/IV	3(100)	0		3(100)	0	
T states			<0.001			0.033
T1	40(33.6)	79		54(45.4)	65	
T2	31(62.0)	19		29(58.0)	21	
T3	6 (85.7)	1		6(85.7)	1	
T4	3(100)	0		3(100)	0	
N states			<0.001			<0.001
N0	51(35.4)	93		63(43.8)	81	
N1	13(81.2)	3		12(75.0)	4	
N2	16(84.2)	3		17(89.5)	2	
Differentiation			0.038			0.416
Well	35(36.1)	62		47(48.5)	50	
Moderate	36(53.7)	31		35 (52.2)	32	
Poor	9 (60.0)	6		10(66.7)	5	

**Table 2:** The positive correlation between YB-1 and MACC1 protein expression in lung adenocarcinoma specimens

	MACC1		P-value
	High	Low	
YB-1			<0.001
High	62	18	
Low	30	69	

Univariate analysis showed that patients with lymph node metastasis (p=0.008), advanced TNM stage (p<0.001), high YB-1 expression (p=0.004) and high MACC1 expression (p=0.044) had poorer survival outcomes (Table 3). As shown in Figure 6B, by Kaplan Meier analysis, the 5-year OS (overall survival) rate of patients with low YB-1 (82%) was significantly higher than those with high YB-1 (53%; p=0.001). Similarly, the 5-year OS rate of lung adenocarcinoma patients with high MACC1 was 61.9%, which was lower than those of patients with low MACC1 (74.3%; p=0.028) (Figure 6C). The multivariate Cox proportional hazards model analysis showed that high TNM stage (Hazard Ratio [HR]=2.369, 95% confidence interval [CI]: 1.110-5.055, p=0.026) and high YB-1 expression (HR=2.638, 95% CI: 1.214-5.733, p=0.014) were independent prognostic risk factors in lung adenocarcinoma (Table 4). Collectively, these data demonstrated that YB-1 was an independent prognostic indicator for lung adenocarcinoma patients.

**Table 3:** Univariate analysis of clinicopathological factors for the five-year overall survival

Variable	5-OS (%)	Log-rank test
		P-value
Sex		0.353
Male	75.5	
Female	62.7	
Age		0.817
≤69y	65.6	
>69y	70.2	
T states		0.098
T1	70.4	
T2	71.0	
T3	33.3	
T4	28.6	
N states		<0.001
N0	75.9	
N1	48.4	
N2	39.9	
TNM stage		<0.001
I/ II	73.6	
III/ IV	32.7	
YB-1		0.001
Low	82	
High	53	
MACC1		0.028
Low	74.3	
High	61.9	

5-OS: five-year overall survival.

**Table 4:** Multivariate analysis of overall survival used Cox-regression

Variable	Multivariate analysis		P-value
	HR	95%CI	
N states			
N0	1.000		
N1/N2	1.200	0.396-3.635	0.747
TNM stage			
I/ II	1.000		
III/ IV	2.369	1.110-5.055	0.026
YB-1			
Low	1.000		
High	2.638	1.214-5.733	0.014
MACC1			
Low	1.000		
High	1.117	0.485-2.571	0.796

### Inhibition of YB-1 suppresses tumor growth in a lung cancer xenograft mouse model *in vivo*

**Figure 7:**
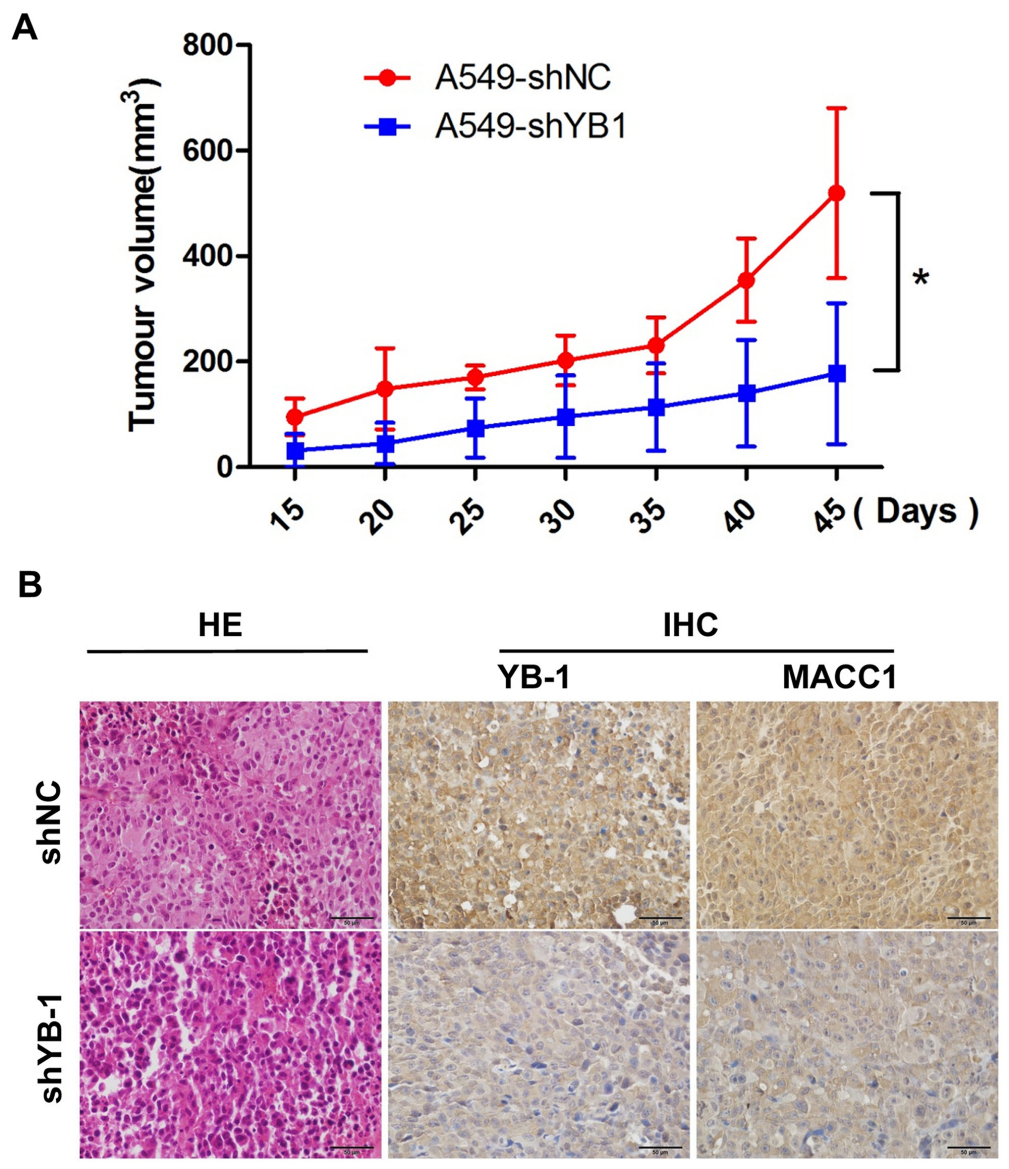
Inhibition of YB-1 suppresses tumor growth in a lung adenocarcinoma xenograft mice model *in vivo* **(A)** The YB-1-silenced A549 cells and their corresponding control cells were simultaneously injected into the right and left flank of the same mice, and mice picture was taken 45 days after injection. Tumor growth curves of subcutaneous implantation models of lung adenocarcinoma are shown. * *P*<0.05. **(B)** H&E staining and immunohistochemical analysis of YB-1 and MACC1 protein expression in tumor samples.

To determine whether YB-1 could be a novel molecular therapeutic target for repressing tumor growth, YB-1-silenced A549 cells and their corresponding control cells were subcutaneously inoculated into BALB/c nude mice. As shown in Figure 7A, YB-1-silenced cells showed weakened tumorigenicity and slower growth than the control cells *in vivo*. The efficacy of YB-1 silencing was confirmed by the marked decrease in the expression of YB-1 in tumor tissues by IHC analysis (Figure 7B). Simultaneously, YB-1 knockdown inhibited the expression of MACC1 in tumor tissues (Figure 7B). These results demonstrated that YB-1 promoted the growth of the xenografted human lung adenocarcinoma through the MACC1 pathway *in vivo* and suggested that YB-1 could be a therapeutic target for lung adenocarcinoma.

## DISCUSSION

Our study provides compelling evidence to suggest that targeting the YB-1/MACC1/c-Met axis could be a potential therapeutic strategy for lung adenocarcinoma patients. Specifically, we report the following novel findings: (1) we found that inhibition of YB-1 diminished cell proliferation, migration and invasion in lung adenocarcinoma cells; (2) for the first time, we demonstrated YB-1 bound to the *MACC1* promoter and directly regulated the MACC1/c-Met signaling pathway; (3) YB-1 was positively correlated with MACC1, and both proteins were over-expressed in lung adenocarcinoma tissues; (4) clinically, YB-1 was an independent prognostic indicator for lung adenocarcinoma patients; and (5) the inhibition of YB-1 suppressed tumor growth in a lung cancer xenograft mouse model *in vivo*.

Numerous studies have shown that YB-1 is over-expressed in various tumor types, including lung cancers, and serves as a novel biomarker of lung cancer progression [24–27]. In the current study, we silenced YB-1 by shRNA in A549 and H1299 lung adenocarcinoma cells. The inhibition of YB-1 diminished cell proliferation, migration and invasion *in vitro*, and suppressed tumor growth in a lung cancer xenograft mouse model *in vivo*. These data indicated that YB-1 is an oncogenic protein, and it could be a potential therapeutic target to suppress the progression of lung adenocarcinoma. The therapeutic inhibition of pathways that block the translational repressor activity of YB-1, such as PI3K-Akt, would also be expected to prevent lung adenocarcinoma growth.

MACC1 was previously reported to mediate invasion and metastasis by regulating HGF/c-Met signaling pathway in several cancer types [19, 28, 29]. We previously reported that MACC1 was a useful marker for predicting postoperative recurrence in patients with lung adenocarcinoma following surgery, and MACC1 promoted lung adenocarcinoma progression [22, 30, 31], the mechanisms underlying its function are poorly understood. In the current study, we analyzed the *MACC1* promoter and found two potential binding sites for the YB-1 protein. Luciferase assay and ChIP assay showed that YB-1 transcriptionally regulated the expression of *MACC1* by directly binding to a specific region of the *MACC1* promoter. These new data reveal a valuable understanding regarding the regulation of MACC1 and provide a new therapeutic target in lung adenocarcinoma.

As the poor prognosis of lung adenocarcinoma is largely associated with late-stage diagnosis [32], novel biomarkers with high sensitivity and specificity are urgently needed to allow the early diagnosis of lung adenocarcinoma and to encourage the development of new treatments. We showed that YB-1 promoted lung adenocarcinoma progression *in vitro* and *in vivo*. In addition, our data revealed that the expression of YB-1 is positively correlated with MACC1 and that both proteins are over-expressed in lung adenocarcinoma tissues, compared to adjacent non-tumor tissues. Consistently, growing evidence indicates that YB-1 and MACC1 could be prognostic predictors for various tumors, including lung cancer [26, 33–35]. In the present study, the 179 patients with lung adenocarcinoma that showed high expression of YB-1 and MACC1 had shorter 5-year overall survival than patients with low expression of YB-1 and MACC1. The multivariate analysis of risk factors showed that high TNM stage and high YB-1 expression, but not MACC1 or the combination of YB-1 and MACC1, were independent prognostic risk factors in lung adenocarcinoma. The unbalanced selection of patients (I stage: n=140; II stage: n=17), specifically, the predominance of early stage patients in our cohort, may have resulted in the failure to detect any statistical significance of MACC1 expression and the combination of YB-1 and MACC1. This will be further studied in our future research.

Collectively, we demonstrated that YB-1 promoted lung adenocarcinoma growth and progression *in vitro* and *in vivo* through directly binding to the *MACC1* promoter and enhancing MACC1/c-Met pathway. Our founding also indicated that YB-1 is an independent prognostic indicator for lung adenocarcinoma patients, and targeting YB-1/MACC1/c-Met axis could be a promising strategy for tumor intervention and therapy.

## MATERIALS AND METHODS

### Cell lines

Human lung cancer cell lines (A549, NCI-H1299, NCI-H358 and NCI-H460), normal human fetal lung fibroblasts cell line (HFL-1) and human bronchial epithelial cells (HBE) were purchased from American Type Culture Collection (ATCC).

### Western blotting

Western blot analysis was conducted using anti-YB-1 (Rabbit, Abcam, ab106579), anti-GAPDH (Mouse, Sigma, A5441), anti-MACC1 (Rabbit, Abcam, ab12148), anti-c-Met (Rabbit, Sangon, AB55219), goat-anti-rabbit IgG conjugated to horseradish peroxidase (HRP) (Thermo, 31460) and goat-anti-mouse IgG conjugated to HRP (Thermo, 31430) which was used as the secondary antibody.

### Transfection of human lung adenocarcinoma cells and generation of stable clones

The shRNA expression vector for silencing YB-1 (pGPU6/GFP/Neo-YBX1- -homo-746 with the target sequence GGTTCCCACCTTACTACAT; pGPU6/GFP/Neo-YBX1-homo-326 with the target sequence AGAAG -GTCATCGCAACGAA) and negative control vector (pGPU6/GFP/Neo-shNC) contain a selectable marker GFP. The stable clones shYB1-1 and shYB1-2 were generated from the pGPU6/GFP/Neo-YBX1-homo-746-transfected A549 cells and pGPU6/GFP/Neo-YBX1-homo-326-transfected A549 cells respectively, and the stable clones shYB1-3 and shYB1-4 were generated from the pGPU6/GFP/Neo-YBX1-homo-746-transfected H1299 cells and pGPU6/GFP/Neo-YBX1-homo-326-transfected H1299 cells respectively.

### MTT assay

Cells were seeded into 96-well plates. After incubation for different time (24h, 48h, 72h), 20μl of MTT solution (5mg/ml, Sigma) was added to each well and cells were incubated at 37°C for another 3.5 hours. The absorbance (OD) was measured at 490nm using a multimode plate reader (Perkin Elmer). Each experimental group contained 6 wells each time.

### Immunostaining

Cells were fixed in 2% paraformaldehyde for 20 min and permeabilized in 0.5% Triton X-100 in PBS for 10 min. The lung adenocarcinoma tissues were dipped into O.C.T. compound (Tissue-Tek), frozen. OCT-embedded tissues were sectioned (6 μM) and placed on glass slides. The sections were fixed with 4% paraformaldehyde for 20 minutes. Slides were incubated with the primary antibody for 60 min. The following antibodies were used: YB-1 (Rabbit, Abcam, ab106579), MACC1 (Mouse, Sigma, AMAB90832) and Ki67 (Mouse, CST, 9449). Immune complexes were stained with the secondary antibody conjugated to Alexa-488 or Alexa-546 (Invitrogen). Nuclei were stained with DAPI (Sigma). Each experimental group contained 6 wells each time, five fields of each well were imaged.

### Wound healing assay

Migration and cell movement throughout the wound area was observed with a phase-contrast microscope after 24 and 48 hours. The percent of the wounded area filled was calculated as follows: [(mean wounded breadth mean remained breadth)/mean wounded breadth]×100(%).

### Migration and invasion assays

Cells (5×10^4^ for Transwell migration assay, 2×10^5^ for Transwell invision assay) were plated on the top side of polycarbonate Transwell filters (Corning; migration assay) or the filter coated with Matrigel (BD; invision assay) in the top chamber of the 24-Well. The cells were incubated at 37 °C for 8 hours (migration assay) or 48 hours (invasion assay). The migrated and invaded cells on the lower membrane surface were fixed in 4% paraformaldedyde for 15 min at RT, air-dried, then stained with 0.5% crystal violet and counted under a microscope (Olympus IX71).

### RNA isolation, reverse transcription and polymerase chain reaction (RT-PCR)

Total RNA was extracted by using TRIzol reagent (Invitrogen) and used to generate cDNA by using PrimeScript RT kit (Takara, RR014A). The amplification was performed in a PCR Thermocycle Instrument. The reaction products were separated by electrophoresis in 2% (w/v) agarose gels. The gels were then scanned and the bands were analyzed using BioRad ChemiDoc XRS+ Imaging System. Quantitative differences between cDNA samples were normalized by including ACTB in all experiments. The PCR primers are listed in Supplementary Table 1.

### Plasmid constructs

The plasmid encoding human YB-1 was generated by PCR amplification and inserted into the pLVX-DsRed-N1-Monomer vector (Clontech). The primers for gene cloning were as follows: YB-1-WT-F 5’-CACTCGAGGCCACCCATGAGCA GCG AGGCCGAGACC-3’; YB-1-WT-R 5’- CATCTA GATTACTCAGCCCCGCCC TGCTCAGCCTCGG-3’.

Promoter of *MACC1* (-2020 to +262) was amplified from A549 cells genomic DNA and inserted into pGL3-Basic (Clontech) to generate *MACC1* promoter-driven luciferase construct pGL3-*MACC1* (-2020 to +262). The site directed mutagenesis was performed using the Quick Change site directed mutagenesis kit (Stratagene) according to the manufacturer's instruction. The pGL3-*MACC1* (-2020 to +262) reporter construct was used as template and a two base pair mutation in the binding sites (Binding site 1: -1860 to -1856; Binding site 2: -1468 to -1464) were inserted using primers with the mutated sequence as underlined in Supplementary Table 1.

pRL-TK Renilla control plasmid was purchased from Clontech.

### Luciferase reporter assays

Cells (1×10^5^) were plated in 24 well plates and transfected with *MACC1* promoter-driven luciferase constructs or control (pGL3-Basic) luciferase constructs and pRL-TK Renilla control plasmid using Lipofectamine 2000. Cells were harvested 24 hours after transfection. Firefly and Renilla luciferase activities were measured using a dual luciferase kit (Promega). The firefly luciferase data for each sample was normalized based on transfection efficiency as measured by Renilla luciferase activity.

### Chromatin immunoprecipitation (ChIP)

ChIP assays were performed according to the manufacturer's protocol (ChIP-IT Express kit, Active Motif). The percentage of bound DNA was quantified against the original DNA input. Specific primers were designed to amplify the *MACC1* promoter sequence which was immunoprecipitated with specific YB1 antibody (Rabbit, Abcam, ab106579). The primers used for the amplification of the precipitated DNA fragments were listed in Supplementary Table 1.

### Patients and specimens

We examined 179 patients with lung adenocarcinoma who underwent radical surgery of the primary tumor and systematic nodal dissection without any adjuvant therapy in the Department of Thoracic Surgery of the First Affiliated Hospital of Dalian Medical University from January 2008 to December 2010. The follow-up of patient were according to our previous process and its ranging from 3 to 60 months after the primary operation (median follow-up time 43.2 months). The study was approved by the Medical Ethical Committees of the First Affiliated Hospital of Dalian Medical University. Characteristics of the patients are presented in Table 1.

### Immunohistochemical staining (IHC)

Resected specimens were deparaffinnized in xylene, rehydrated and incubated in 3% (v/v) hydrogen peroxide (Sigma-Aldrich) for 10 minutes. Antigenic retrieval was processed with sodium citrate. IHC staining was performed using Streptavidin-Peroxidase IHC assay kit (ZSGB-bio, China) following the manufacturer's instructions. Antibodies of YB-1 and MACC1 antibody diluted 1:200 and 1:100 in PBS containing 2% goat bovine serum respectively. Immunostaining was evaluated by two pulmonary pathologists using a blind protocol design. For each specimen, the total score of intensity expression (negative staining: 0 point; weak staining: 1point; moderate staining: 2 point; and strong staining: 3 point) multiplying stained cell numbers (positive cells as ≤25% of the cells: 1 point; 26-50% of the cells: 2 point; 51-75% of the cells: 3 point; >75% of the cells: 4 point) of YB-1 or MACC1 was estimated. When the sample was scored ≥6 point, we defined it as high expression, otherwise low expression.

### Mouse xenograft assay

BALB/c athymic nude mice (4-6 weeks of age) were used, and each experimental group consisted of 6-10 mice. Briefly, A549 cells (2×10^6^) were injected subcutaneously into the right and left flank of the same mice. Tumors were measured perpendicular dimensions using calipers. Volumes were estimated using the formula (α^2^×β)/2, where α is the shorter of the two dimensions, and β is the longer one. Tumor specimens were fixed in formalin and embedded in paraffin.

### Statistical analysis

Student's t-test or analysis of variance (ANOVA) was used to compare the values of the test and control samples *in vitro* and *in vivo*. The associations between YB-1 or MACC1 expression and categorical variables were compared by Pearson chi-square test. Survival curves were calculated using the Kaplan Meier method. The log-rank test was used to analyze overall survival (OS) time between different clinicopathological factors in lung adenocarcinoma. Multivariate analysis was performed using the Cox regression model. Data was analyzed by the SPSS 20 software (Inc, Chicago, IL). Values of p<0.05 were considered statistically significant difference.

## SUPPLEMENTARY MATERIALS FIGURES AND TABLE



## Abbreviations

YB-1: Y-box-binding protein-1
MACC1: metastasis associated in colon cancer-1
NSCLC: non-small-cell lung cancer
ChIP: chromatin immunoprecipitation
IHC: immunohistochemistry
