# Correction: YB-1 regulates tumor growth by promoting MACC1/c-Met pathway in human lung adenocarcinoma

**DOI:** 10.18632/oncotarget.28393

**Published:** 2023-03-31

**Authors:** Tao Guo, Shilei Zhao, Peng Wang, Xiaoyuan Xue, Yan Zhang, Mengying Yang, Nan Li, Zhuoshi Li, Lingzhi Xu, Lei Jiang, Lei Zhao, Patrick C. Ma, Rafael Rosell, Jinxiu Li, Chundong Gu

**Affiliations:** ^1^Department of Thoracic Surgery, The First Affiliated Hospital of Dalian Medical University, Dalian 116011, China; ^2^Lung Cancer Diagnosis and Treatment Center of Dalian, The First Affiliated Hospital of Dalian Medical University, Dalian 116011, China; ^3^Institute of Cancer Stem Cell, Dalian Medical University, Dalian 116044, China; ^4^Department of Radiation Oncology, Qianfoshan Hospital Affiliated to Shandong University, Jinan 250000, China; ^5^The Second Affiliated Hospital, Dalian Medical University, Dalian 116011, China; ^6^The Fourth Affiliated Hospital, Anhui Medical University, Hefei 230000, China; ^7^Aerodigestive Oncology Translational Research THOR, Department of Solid Tumor Oncology, Taussig Cancer Institute, Cleveland Clinic, Cleveland, OH 44195, USA; ^8^Breakthrough Cancer Research Unit, Pangaea Biotech, Dexeus University Institute, Catalan Institute of Oncology, Badalona 08916, Spain; ^ ^Written on behalf of the AME Thoracic Surgery Collaborative Group; ^*^These authors have contributed equally to this work

**This article has been corrected:** In Figure 2D, the ‘shYB1-3’ panel contains an accidental overlap of the ‘shYB1-3’ panel in Figure 3B. The corrected Figure 2D, produced using the original data, is shown below. The authors declare that these corrections do not change the results or conclusions of this paper.

Original article: Oncotarget. 2017; 8:48110–48125. 48110-48125. https://doi.org/10.18632/oncotarget.18262

**Figure 2:**
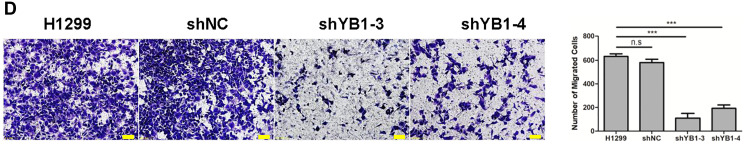
Inhibition of YB-1suppresses migration of lung adenocarcinoma cells. (**A**, **B**) Wound healing assay of lung adenocarcinoma cells in the YB-1-silenced cells (shYB1-1 and shYB1-2: YB-1-silenced A549 cells; shYB1-3 and shYB1-4: YB-1-silenced H1299 cells) and their corresponding control cells. The distance from the wound edge was calculated with corresponding summary (right panel). (**C**, **D**) Transwell assay in lung adenocarcinoma cells in the YB-1-silenced cells and their corresponding control cells. Columns were averaged from three independent experiments. ^*^ *P *< 0.05, ^***^ *P *< 0.01, ^***^ *P *< 0.005, two-tailed Student’s *T*-tests. Error bars represent mean ± s.d. Scale bars, 100 μm (A, B), 50 μm (C, D).

