# Supplementary material for: YB-1 regulates tumor growth by promoting MACC1/c-Met pathway in human lung adenocarcinoma
Source: Oncotarget. 2017 May 29;8(29):48110–25. doi: 10.18632/oncotarget.18262 (PMC5564630; doi:10.18632/oncotarget.18262)
Supplement: Supplementary file 1 [file oncotarget-08-48110-s001.pdf]

YB-1 regulates tumor growth by promoting MACC1/c-Met pathway in human lung adenocarcinoma

SUPPLEMENTARY INFORMATION

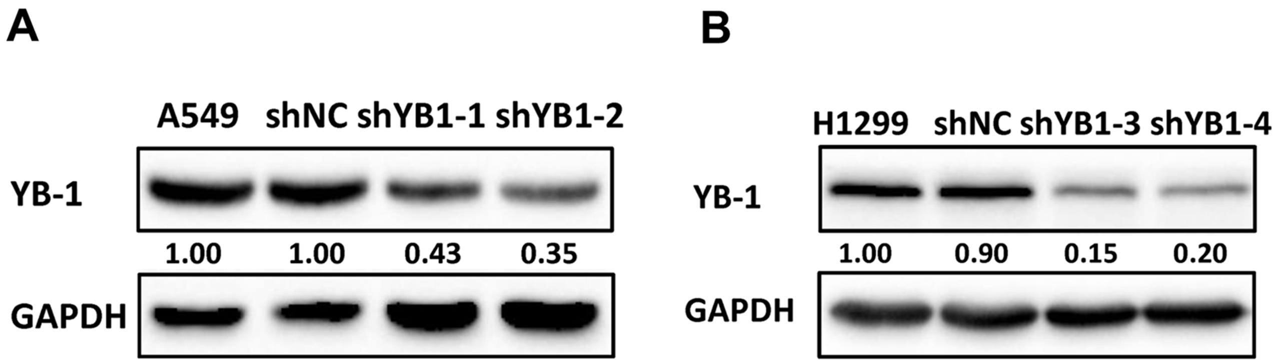

Supplementary Figure 1: Efficiencies for knockdown of YB-1 were tested by western blot analysis. The YB-1-silenced A549 cell clones (shYB1-1, shYB1-2) **(A)** and YB-1-silenced H1299 cell clones (shYB1-3, shYB1-4) **(B)** were selected after transfection using G418 (800 mg/L), then these stable cell lines were analyzed by Western Blotting assay to confirm the expression level of YB-1.

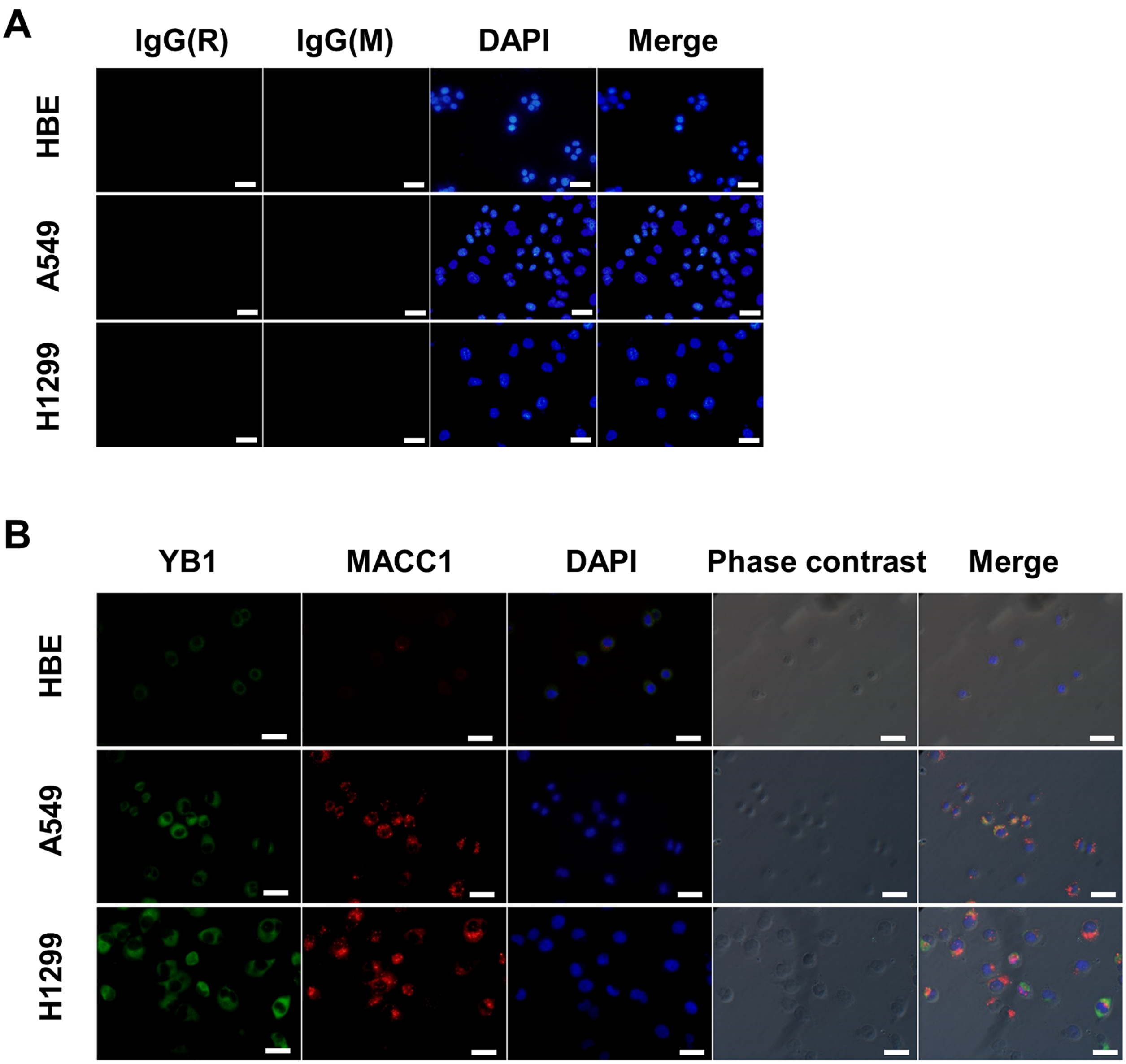

Supplementary Figure 2: Co-localization of YB-1 and MACC1 in HBE, A549 and H1299 cells by immunofluorescence assay. (A) The control images of Figure 5A. IgG (R): Rabbit IgG was used as the primary antibody; IgG (M): Mouse IgG was used as the primary antibody. **(B)** Immunofluorescence assay in HBE cells, A549 cells and H1299 cells. The results included companion phase contrast images were shown. Green: YB-1; Red: MACC1. Scale bars, 20 µm.

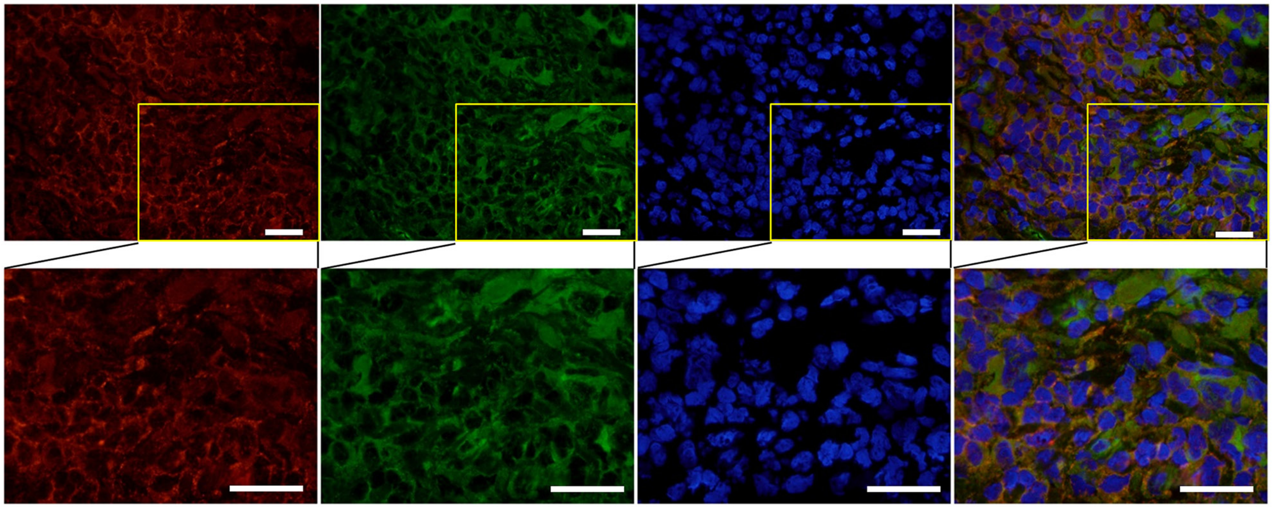

Supplementary Figure 3: Co-localization of YB-1 and MACC1 in lung adenocarcinoma tissues by immunofluorescence assay. Lung adenocarcinoma tissues were collected, frozen in O.C.T. compound, and sectioned. Slides were stained with primary YB-1(red), MACC1 (green) antibody and DAPI. Representative images of cells from lung adenocarcinoma tissues are shown. Scale bars, 20 µm.

Supplementary Table 1: Sequences for primers

Identifier type	Sense sequence(5'-3')	Antisense sequence(5'-3')
**Primers used for PCR**		
YB-1	ACCACAGTATTCCATCCCTCCTG	ATCTTCTTCATTAGCCGTCCTCTC
MACC1	TTCTTTTGATTCCTCCGGTGA	ACTCTGATGGGCATGTGCTG
ACTB	CATGTACGTTGCTATCCAGGC	CTCCTTAATGTCACGCACGAT
**Primers used for generation of the MACC1 promoter luciferase constructs**		
pGL3-MACC1 (-2020 to +262)	TTACGCGTGCCCGTAGATTAGATAA	AACTCGAGGCCCACAGAATACCAGTTACC
pGL3-MACC1-mutant1 (-1860 to -1856)	AAGTGCACAGCAGGGAGGGGAGCTGGTAAACAGTG	CACTGTTTACCAGCTCCCCTCCCTGCTGTGCACTT
pGL3-MACC1-mutant2 (-1489 to -1485)	ACACACCCATGTAAACCGGTGAAATGAGATATACA	TGTATATCTCATTTCACCGGTTTACATGGGTGTGT
**Primers used for amplifying the MACC1 promoter sequence in ChIP assay**		
YB-1 binding site1	GGAGAAGGGATCCATAAATGC	ATTGAACCCTTGACAGTACCC
YB-1 binding site2	TAGGGATACAATGAGGCCTTGC	TCTGCATGGCATTTCTCCTCC
